# Heparan Sulfates Regulate Vascular Reactivity in Syndecan 1 Knockout Mice

**DOI:** 10.3390/ijms27031386

**Published:** 2026-01-30

**Authors:** Simone R. Potje, Aishwarya Katiki, Paulo W. Pires, Andreia Zago Chignalia

**Affiliations:** 1 Department of Medical Sciences, Minas Gerais State University, Passos 37900-106, MG, Brazil; simone.potje@uemg.br; 2 Department of Anesthesiology, College of Medicine—Tucson, University of Arizona, Tucson, AZ 85724, USA; akatiki@uchicago.edu; 3 Department of Physiology, College of Medicine—Tucson, University of Arizona, Tucson, AZ 85724, USA; ppires@arizona.edu; 4 Department of Pharmacology and Toxicology, College of Pharmacy—Tucson, University of Arizona, Tucson, AZ 85724, USA; 5 Department of Internal Medicine, College of Medicine—Phoenix, University of Arizona, Phoenix, AZ 85004, USA

**Keywords:** syndecan 1, glypican 1, heparan sulfate, heparinase III, vascular reactivity

## Abstract

Heparan sulfates (HS) are polysaccharides abundantly expressed in the extracellular matrix and the glycocalyx of endothelial cells, having a putative role in vascular function. The role of HS in vascular reactivity remains unclear. Herein, we sought to determine whether HS regulate the vascular tone in physiological conditions. Using male, 6–8-weeks-old, CD1, C57BL/6, syndecan 1 (*Sdc1^−/−^*) and glypican 1 (*Gpc1^−/−^*) knockout mice, we investigated if the degradation of HS with heparinase III altered vascular reactivity to norepinephrine (NE), acetylcholine (ACh) and potassium chloride (KCl). Our findings indicate that HS are crucial players in the vascular response to NE and ACh in CD1, C57BL/6, and *Sdc1^−/−^* but not in *Gpc1^−/−^* mice. Both *Sdc1^−/−^* and *Gpc1^−/−^* showed increased compensatory expression of syndecan 2 and syndecan 4. However, while *Sdc1^−/−^* showed decreased expression of glypican 1, *Gpc1^−/−^* showed increased expression of syndecan 1 in aortic homogenates. The lack of response to the vascular reactivity effects of heparinase III in *Gpc1^−/−^* suggests a differential role of HS to proteoglycan function in the regulation of the vascular tone. Our data demonstrate a physiological role for HS in the regulation of the vascular tone in physiological conditions.

## 1. Introduction

Heparan sulfates (HS) are linear polysaccharides heavily expressed in the extracellular matrix and the endothelial glycocalyx where they covalently bind to proteoglycans, forming heparan sulfate proteoglycans (HSPGs), macromolecules with emerging role in the regulation of vascular function. More specifically, HS predominates in the vasculature, accounting for approximately 50 to 90% of glycosaminoglycans (GAGs) binding to endothelial proteoglycans (Reitsma et al., 2007 [1]).

The membrane-bound HSPGs Syndecan 1 (Sdc1) and Glypican 1 (Gpc1) are known to play a key role in vascular homeostasis by regulating endothelial function. Syndecans and Glypicans carry HS covalently attached to the extracellular domains. The functions of Sdc1 include the following: (I) mechanosensing [2], (II) regulation of vascular permeability [3], (III) modulation of vascular smooth muscle cells (VSMC) differentiation [4], and (IV) a biomarker of vascular inflammatory processes [5,6]. On the other hand, Gpc1 prevents endothelial dysfunction [7], acts as the main mediator of flow-induced nitric oxide (NO) production [8], regulates growth factor signaling and angiogenic pathways [9,10], and is required for vascular contractility in response to norepinephrine (NE) [11].

A key biophysical feature of Sdc1 and Gpc1 is that they show a mobile extracellular domain that facilitates interaction with neighboring membrane proteins. Importantly, such interactions are thought to be mediated by HS chains covalently bound to their extracellular domain, implicating HS in possible biological functions of HSPGs [12].

It is currently known that HS play a key role in vascular homeostasis, as they are involved in the production of NO, a biomarker of endothelial function [13]. Moreover, decreased HS content is associated with inflammatory and oxidative processes that underlie cardiovascular diseases [14,15]. The role of HS in the regulation of vascular tone in physiological conditions is unresolved.

Previous studies from our group showed that Sdc1 and Gpc1 regulate endothelial-dependent vasodilation by mechanisms associated with NO signaling. Notably, the mechanisms whereby Sdc1 and Gpc1 regulate vasoconstriction in mice differ. Our previous studies showed that syndecan 1 knockout mice (*Sdc1^−/−^*) show endothelial-dependent impaired vasoconstriction [11]. Whether HS play a role in the effects of Sdc1 and Gpc1 on the regulation of the vascular tone is unexplored. Herein, we sought to investigate if HS regulate vascular tone and contribute to the vasoactive effects of Sdc1 and Gpc1 in physiological conditions.

## 2. Results

### 2.1. Heparinase III Impaired Vasoreactivity in Sdc1^−/−^ and C57BL/6 Aortic Rings

**Figure 1:**
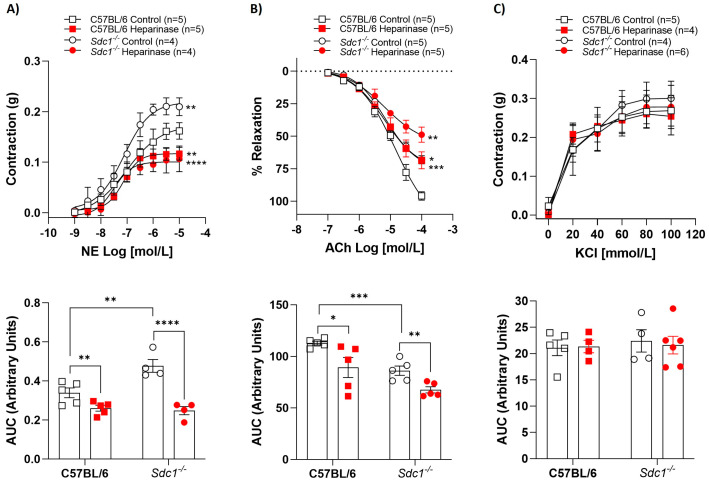
Heparinase III impaired vasoreactivity in *Sdc1^−/−^* and C57BL/6 aortic rings. Concentration-response curves for (**A**) norepinephrine (NE, 1 nmol/L to 10 μmol/L), (**B**) acetylcholine (ACh, 0.1 μmol/L to 0.1 mmol/L), and (**C**) potassium chloride (KCl, 20–100 mmol/L) were performed in the absence (Control) and presence of heparinase III (15 mU/mL, 2 h) in aortic rings with endothelium from *Sdc1^−/−^* and C57BL/6 mice. The area under the curve (AUC, in arbitrary units) was calculated from the concentration-effect curves. Data represent the mean ± SD of the experiments, and n = 4–6 represents the number of aortic rings used in the experiments. * *p* < 0.05, ** *p* < 0.01, *** *p* < 0.001, **** *p* < 0.0001 statistical difference in maximum effect values or AUC values between C57BL/6 Heparinase vs. C57BL/6 Control, or *Sdc1^−/−^* Heparinase vs. *Sdc1^−/−^* Control, or *Sdc1^−/−^* Control vs. C57BL/6 Control. (Two-way ANOVA followed by Tukey’s post hoc test).

Vascular reactivity was assessed in isolated aortic rings with intact endothelium. Heparinase III reduced the NE-stimulated contractile effect, as well as impaired ACh-evoked relaxation effect in aortic rings from C57BL/6 and *Sdc1^−/−^* mice compared with control (non-treated) rings (Figure 1A,B). Likewise, a reduction in area under the curve (AUC) analyses in the presence of heparinase III was observed in the vasoconstriction response to NE and in the vasodilation response to ACh. However, heparinase III did not change the KCl-stimulated vasoconstriction in aortic rings from C57BL/6 and *Sdc1^−/−^* or AUC analyses (Figure 1C).

### 2.2. Heparinase III Does Not Change Vasoreactivity in Gpc1^−/−^

**Figure 2:**
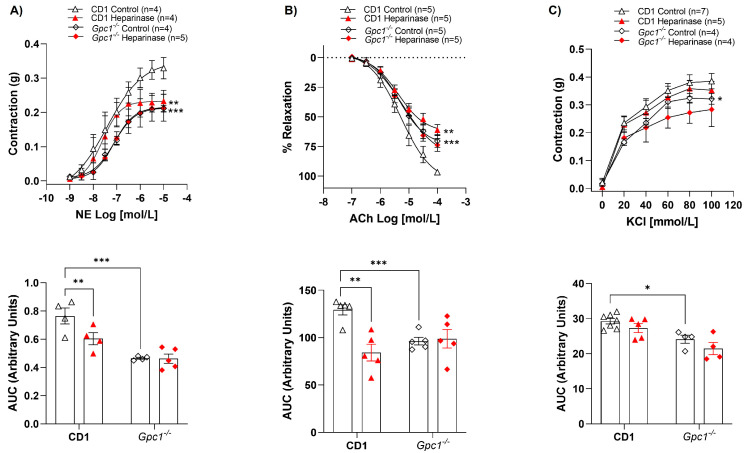
Heparinase III does not change vasoreactivity in *Gpc1^−/−^*. Concentration-response curves for (**A**) norepinephrine (NE, 1 nmol/L to 10 μmol/L), (**B**) acetylcholine (ACh, 0.1 μmol/L to 0.1 mmol/L), and (**C**) potassium chloride (KCl, 20–100 mmol/L) were performed in the absence (Control) and presence of heparinase III (15 mU/mL, 2 h) in aortic rings with endothelium from *Gpc1^−/−^* and CD1 mice. The area under the curve (AUC, in arbitrary units) was calculated from the concentration-effect curves. Data represent the mean ± SD of the experiments, and n = 4–7 represents the number of aortic rings used in the experiments. *p < 0.05, ** *p* < 0.01, *** *p* < 0.001, statistical difference in maximum effect values or AUC values between CD1 Heparinase vs. CD1 Control, or *Gpc1^−/−^* Control vs. CD1 Control. (Two-way ANOVA followed by Tukey’s post hoc test).

While heparinase III reduced NE-mediated contraction (Figure 2A) and ACh-induced relaxation (Figure 2B) in CD-1 aortic rings, heparinase III did not interfere with the contractile or relaxant response of these agonists (Figure 2A,B) in *Gpc1^−/−^* aortic rings. Analysis of the AUC showed that heparinase III decreased the vasoconstrictive response to NE by approximately 23% and the ACh-induced vasodilation by 40% in CD1 mice. No changes in AUC were observed in *Gpc1^−/−^* aortic rings. KCl-stimulated vasoconstriction was similar in the absence and presence of heparinase III in aortic rings from CD1 and *Gpc1^−/−^* (Figure 2C). Likewise, no differences were seen in vascular response to heparinase via AUC analyses in all groups.

### 2.3. Glycocalyx Modifications in Gpc1^−/−^ and Sdc1^−/−^ Mice

**Figure 3:**
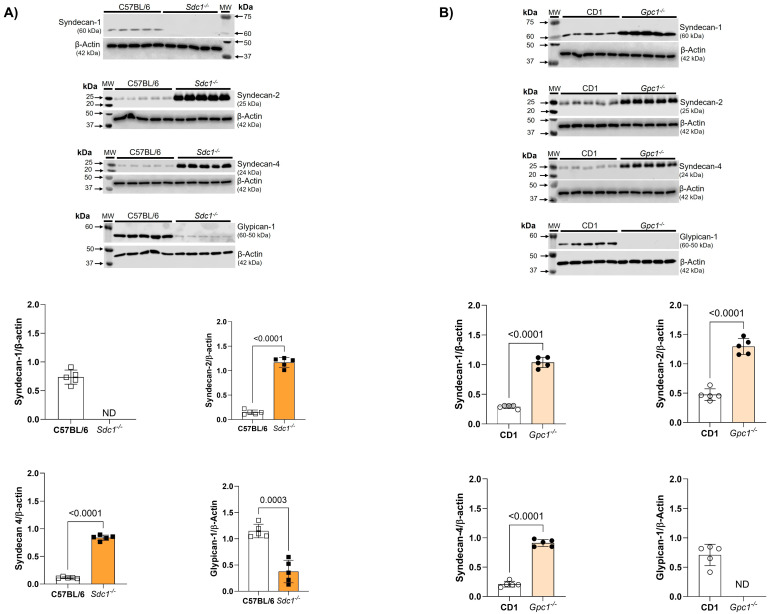
Increased syndecans expression in *Gpc1^−/−^* and *Sdc1^−/−^*. The expression of syndecan 1, syndecan 2, syndecan 4, and glypican 1 was analyzed in aortic rings of *Sdc1^−/−^* and *Gpc1^−/−^* and compared to their respective wild-type controls (C57BL/6 and CD1). (**A**) The expression of syndecan 2, syndecan 4 are increased and the expression of glypican 1 is decreased in aortic rings of *Sdc1^−/−^* mice. (**B**) The expression of syndecan 1, syndecan 2 and syndecan 4 are increased in aortic rings of *Gpc1^−/−^* mice. Changes in brightness and contrast were equally applied in all representative Western blot images to better visualize the bands. The β-actin loading control shown here for Syndecan-4 is the same as previously published in Potje et al., 2021 [11]. This happened because the target proteins were detected on the same membrane that was cut into specific molecular weight ranges. Three targets were probed [α1D (40-60KDa), α2B (62KDa) and syndecan 4 (24Kda)] and normalized to the β-actin of that same membrane. Data represent the mean ± SD of the experiments, and n = 5 represents the number of samples or aortic rings used in the experiments.

We confirmed the deletion of Sdc1 and Gpc1 in our knockout lines by immunoblotting. As expected, Sdc1 expression was absent in *Sdc1^−/−^* aortas. This was associated with the reduced expression of Gpc1 and increased expression of Syndecan 2 (Sdc2) and Syndecan 4 (Sdc4) when compared with C57BL/6 aorta samples (Figure 3A). Additionally, we confirmed the absence of Gpc1 in *Gpc1^−/−^* aorta homogenates. This was associated with the increased expression of Sdc1, Sdc2, and Sdc4 when compared to CD1 aorta samples (Figure 3B).

### 2.4. Impaired Endothelium-Dependent Relaxation Is Associated with Decreased Akt Activity in Sdc1^−/−^ and Gpc1^−/−^ Aortic Rings

**Figure 4:**
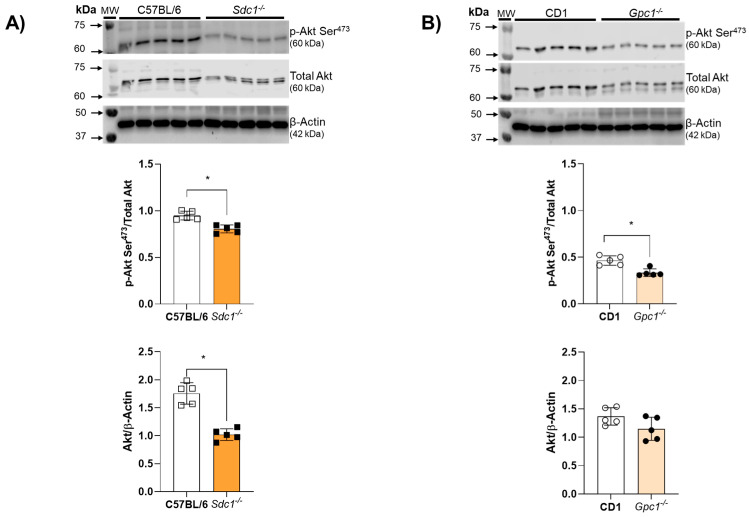
Impaired Akt/NOS signaling in *Sdc1^−/−^* and *Gpc1^−/−^*. (**A**,**B**) Representative images of pS^473^-Akt and total Akt in aorta samples from *Sdc1^−/−^* and *Gpc1^−/−^* were compared to their respective wild-type controls (C57BL/6 and CD1). Data represent the mean ± SD of the experiments and n = 5 represents the number of samples or aortic rings used in the experiments. * *p* < 0.05 statistical difference between *Sdc1^−/−^* vs. C57BL/6, *Gpc1^−/−^* vs. CD-1 (Student’s *t*-test).

Akt activity was assessed by the ratio between Akt phosphorylation at Serine^473^ residues and total Akt expression. Akt activity was reduced in aortic rings from *Sdc1^−/−^* and *Gpc1^−/−^* mice (Figure 4A,B). Total Akt expression was decreased in aortic rings from *Sdc1^−/−^* mice compared to C57BL/6 but remained unchanged between *Gpc1^−/−^* and CD1 mice (Figure 4B).

## 3. Discussion

In this study, we show unprecedented findings that HS has a physiological vasoactive function, thus modulating vascular reactivity. Notably, the global genetic deletion of Sdc1, but not Gpc1, compromises the vasoactive role of HS. This suggests different implications of HS on proteoglycan vascular function.

To assess the contribution of the predominant glycosaminoglycan, namely HS, to vascular reactivity, we used heparinase III, which is a pharmacological tool that cleaves biologically active HS into inactive fragments, such as disaccharides [16]. Heparinase III is a polysaccharide lyase that cleaves heparan sulfate chains (Dong et al., 2012 [16]), and there are studies demonstrating its action in different vascular beds. Porcine femoral artery treated with heparinase III (15 mU/mL, 37° C, 2 h) demonstrated impairment of the vasodilation and NO production (Kumagai et al., 2009 [17]). In addition, heparinase III (~0.2 mU/μL, 37° C, 45 min) impairs intraluminal flow-mediated vasodilation in murine mesenteric arteries (Cho et al., 2022 [18]). Thus, we use heparinase III as a pharmacological tool to study the role of HS in vascular function.

We demonstrated that HS degradation reduces NE-mediated vasoconstriction and impairs endothelium-mediated vasodilation in aortic rings from wild-type (C57BL/6 and CD1) and *Sdc1^−/−^* mice, confirming that HS modulate vascular function. However, HS degradation did not alter the vascular noradrenergic contractile or relaxant response in aortic rings from *Gpc1^−/−^* animals, suggesting that HS modulate vascular reactivity differently in *Sdc1^−/−^* and *Gpc1^−/−^* mice. Previous studies from our group have investigated the effects of NE, ACh, and KCl in the vascular reactivity of *Sdc1^−/−^* and *Gpc1^−/−^* aortic rings [11]. The control curves (without heparinase III) in the present study are generated from new control experiments performed to specifically investigate the role of heparinase III in aortic vascular reactivity. The new control experiments shown in this study replicate our previous findings.

To better understand the data presented in the concentration-response curves for contraction or relaxation, it is important to note that there was the compensatory expression of different proteoglycans in the genetically modified mice used in this study. Specifically, *Sdc1^−/−^* mice show increased Sdc2 and Sdc4 and decreased Gpc1 in aorta homogenates. This corroborates previous reports that showed a thinner but hydrodynamically relevant glycocalyx in *Sdc1^−/−^*. The authors suggested that to adapt to the absence of Sdc1, an increase in the expression of other proteoglycans could occur [19], as observed in our data.

Regarding the genetic deletion of Gpc1, we observed an overexpression of Sdc1, Sdc2 and Sdc4 in *Gpc1^−/−^* aorta homogenates. This pattern of proteoglycan expression differs from the pulmonary pattern of proteoglycan expression previously reported by our group [20]. This may relate to the different cell types that constitute the lungs and the aorta or from the already existing differences in glycocalyx thickness and composition natural from different vascular beds [21]. As the glycocalyx is ubiquitously expressed in all cells of the body, it is unclear if the changes in proteoglycan expression are limited to the endothelial cells or if other layers of the blood vessel are driving the changes in proteoglycan content seen in the present study. Based on our findings that suggest modifications of the glycocalyx in the aortas, we predict that there was a redistribution of HS chains in *Sdc1^−/−^* and *Gpc1^−/−^* aortas.

Although HS chains have a similar structure characterized by iduronate-rich S domains and *N*- and *O*-sulfation alternating with *N*-acetylated sequences, syndecans and Gpc1 have been shown to exhibit reproducible differences in contiguous *N*-sulfated sequences. Specifically, syndecans have 23% *N*-sulfated disaccharides, and Gpc1 has 32% *N*-sulfated disaccharides, i.e., Gpc1 exhibits an increased contiguously *N*-sulfated region [22]. Furthermore, it has been demonstrated that heparinase III acts predominantly in HS regions with a lower degree of sulfation [23]. Based on this information, heparinase would have a predominant action on syndecans because they present less sulfated regions. Even though both *Sdc1^−/−^* and *Gpc1^−/−^* show the expression of Sdc2 and Sdc4, no changes in vascular reactivity were observed in *Gpc1^−/−^* aortic rings. We consider that HS may regulate the vasoactive role of Gpc1 and hypothesize that the overexpression of syndecan isoforms may occur in other cell types that do not interfere with endothelial-dependent vascular reactivity.

The effects of heparinase III have different magnitudes according to mice strain. As noted in the results section, heparinase III decreased the effects of NE in aortic vasoconstriction in approximately 50% in *Sdc1^−/−^* mice, but only 20% in wild-type mice. This suggests that Sdc1, as a major constituent of the endothelial glycocalyx, may serve as a buffer to the effects of heparinase III or that heparinase III shows increased affinity to other glycocalyx components according to sulfation. On the other hand, heparinase III decreases the vasodilatory response to ACh by 40% in CD1 and 20% in C57BL/6 mice, showing a genetic variability in the effects of this drug. How the genetic background may interfere with the potency of heparinase is unknown, and this will be a future direction of investigation for our group.

Previously, we demonstrated impaired endothelium-dependent relaxation in *Sdc1^−/−^* and *Gpc1^−/−^* aortas. This was associated with reduced endothelial nitric oxide synthase (eNOS) activity and decreased NO production [11]. Herein, we replicated the concentration-response curves for ACh in aortic rings from other groups and confirmed the impaired vasodilatory previously reported by our group. To further investigate the mechanism of impaired endothelial vasorelaxation, we expanded our molecular studies and questioned whether Akt, a known upstream regulator of eNOS [24], was dysregulated in these mice. Our findings show that Akt activity is reduced in both *Sdc1^−/−^* and *Gpc1^−/−^*, corroborating our previous studies in lung endothelial cells [20]. Thus, we confirmed that the genetic deletion of two different proteoglycans (Sdc1 and Gpc1) compromises endothelial function and impairs the Akt/eNOS signaling pathway. Although other endothelium-derived factors may contribute to vasodilation, it is known that in large conductance arteries, NO is the main endothelial-derived vasodilator factor produced in response to acetylcholine. Other endothelial-derived factors such as EDHF play a significant role in small resistance arteries. Thus, the effects of heparinase III on ACh-induced vasodilation are expected to be associated with decreased NO levels.

To further explore the role of HS in endothelial reactivity, we used heparinase III as a pharmacological tool to degrade HS. Herein, we show that heparinase III impaired the endothelium-dependent vasodilatory response in C57BL/6, CD1, and *Sdc1^−/−^* aortic rings. These findings support previous studies implicating HS in NO production and endothelial function. Specifically, the enzymatic removal of HS with heparinase III inhibited NO production induced by steady shear stress (20 dyn/cm^2^) in isolated bovine aortic endothelial cells (BAECs). However, the cells were still able to produce NO in response to bradykinin after heparinase treatment [25]. In addition, vasodilation and NO production were reduced in the porcine femoral artery after heparinase III treatment [17]. Furthermore, intraluminal flow-mediated vasodilation was impaired after heparinase III-induced HS disruption in murine resistance arteries [18]. Moreover, genetically modified mice overexpressing heparanase, the only mammalian enzyme known to degrade HS, showed increased arterial thickness, cell density, and mechanical compliance, indicating endothelial dysfunction [26]. These results demonstrate that HS partially contributes to the production of NO, thus acting directly in vascular homeostasis.

Damage to the endothelial glycocalyx caused by heparinase III reduced NE-induced contraction in C57BL/6, CD1, and *Sdc1^−/−^* aortic rings. We had previously reported that *Gpc1^−/−^* showed impaired vasoconstrictive response to NE [11]. Thus, the lack of heparinase III effects on *Gpc1^−/−^* aortic rings may simply reflect the underlying impaired vasoconstrictive response already described in *Gpc1^−/−^* mice. Heparinase III can impair muscle cell proliferation and can also influence the activity of receptors involved in vascular smooth muscle contraction, thus affecting vasoconstriction. Heparinase III has been shown to limit the proliferation of rat VSMC in vitro and in vivo by compromising the binding of HS to growth factors that stimulate arterial proliferation [27]. It is known that HS are negatively charged linear polysaccharide structures, which are also called polyanions. Dasso and Taylor [28] demonstrated in liver tissue that three different polyionic compounds (heparin, trypan blue, or suramin) were able to prevent the formation of the high-affinity adrenaline-receptor-G protein complex; however, there was no interference with the binding to the antagonist. Thus, this reduction in the NE-induced vasoconstrictor response caused by heparinase may be associated in part with impaired G-protein-receptor complex formation. We ponder it is unlikely that heparinase III reduced muscle cell proliferation in our experiments as the impaired vasoreactivity was seen only 120 min after the incubation with heparinase III, an insufficient period to alter cell proliferation in isolated rings ex vivo.

High concentrations of potassium ions can directly alter the membrane potential of VSMC, promoting depolarization and, consequently, vasoconstriction, which is subsequently reversed after removal of the solution [29]. We previously demonstrated that KCl-induced contraction was similar in aortic rings with and without endothelium from C57BL/6 and *Sdc1^−/−^* mice. However, KCl-evoked contraction was reduced in intact and endothelium-depleted aortic rings from *Gpc1^−/−^* compared to CD1. This was associated with decreased phosphorylation of the IP3 receptor and confirmed that Gpc1 is required for IP3 signaling and contraction in VSMC [11]. In this study, we demonstrated that the concentration-response curves to KCl were similar in the absence or presence of heparinase for all groups. This suggests that heparinase III does not interfere with the IP3-calcium-dependent contraction pathway. Thus, the effects of HS in the contractile response to NE are restricted to the endothelial layer.

Taken together, our data demonstrate that HS contributes to the regulation of vascular tone with a predominant effect on endothelial function, with an important decrease in NE-induced constriction. Studies with animal models of disease (such as essential hypertension, catecholaminergic surge or shock) are required to examine whether targeting HS with heparinase III shows beneficial effects towards vascular diseases. More experiments are needed to understand the role of HS and heparinase in vascular homeostasis.

Limitations of the study. This study was conducted only with male mice. Whole system physiology shows the complexity of a unified response toward HS degradation. As multiple proteoglycans carry HS, the effects of heparinase III will not be selective to syndecans and glypicans. Our current scientific question is to dissect the mechanisms on how membrane-bound HSPGs can actively signal to regulate vascular reactivity. Previous work from our group described impaired endothelium-dependent vasodilation in *Sdc1^−/−^* and *Gpc1^−/−^* and impaired NE-induced vasoconstriction only in *Gpc1^−/−^*. Whether the impaired signaling mechanisms observed in these knockout mice result from the core proteins or its covalently attached HS content was not previously explored. Thus, this work was focused on addressing if HS were key players in the impaired vascular reactivity in *Sdc1^−/−^* and *Gpc1^−/−^* mice. It is possible that other proteoglycans and HS not bound to Sdc1 and Gpc1 play a role in the vascular reactivity changes observed in the present study. We ponder that the compensatory expression of other Sdc isoforms can mask some vascular responses. These limitations will be addressed in future studies. Although observations of impaired endothelial function correlate to glycocalyx modifications, molecular studies are needed to further pinpoint the exact role of HS to the biological role of Sdc1 and Gpc1 towards vascular reactivity. This represents a future direction of study to our group.

## 4. Materials and Methods

### 4.1. Drugs

Chemicals were purchased at the highest purity available. Acetylcholine (ACh), Norepinephrine bitartrate (NE), Potassium Chloride (KCl), and Heparinase III were purchased from Sigma Aldrich (Burlington, MA, USA). Antibodies were as follows: Anti-Syndecan-1 (#sc-7099), Anti-Syndecan 2 (#sc-376160), Anti-Syndecan 4 (#sc-33912), Anti-pAkt (#sc-7985) and Anti-Akt (#sc-55523) were purchased from Santa Cruz Biotechnology (Dallas, TX, USA); Anti-Glypican-1 (#MAB2600) was purchased from EDM Millipore (Burlington, MA, USA) anti β-actin was purchased from Cell Signaling (Danvers, MA, USA) (#4970).

### 4.2. Animals

In this study, we used male glypican 1 knockout (*Gpc1^−/−^*), syndecan 1 knockout (*Sdc1^−/−^*), CD1, and C57BL/6 mice at 6 to 8 weeks of age. CD1 and C57BL/6 were the genetic background controls for *Gpc1^−/−^* and *Sdc1^−/−^*, respectively. A breeding pair of *Gpc1^−/−^* mice was kindly donated by Dr. Arthur Lander (University of California, Irvine, CA, USA). Dr. Pyong Park (Children’ s Hospital, Boston, MA, USA) kindly donated a breeding pair of *Sdc1^−/−^* mice. CD1 and C57BL/6 wild-type mice were purchased from Charles River Laboratories (Wilmington, MA, USA). All animal studies were approved by The University of Arizona (18–491) Animal Committee. These studies were conducted according to the Guide for the Care and Use of Laboratory Animals published by the National Institutes of Health.

### 4.3. Genotyping

*Gpc1^−/−^* and *Sdc1^−/−^* tail tips were cut, allocated in different tubes, and digested overnight (50–60 °C) with proteinase K solution (20 mg/mL, code 25530049, Invitrogen, Carlsbad, CA, USA). The following day, 250 μL of NaCl (6 mol/L) was added to each tube. The tissue was homogenized and immediately centrifuged (10,000 rpm, 10 min, 4 °C). The supernatant was collected and placed in a clean tube where 650 μL of isopropanol was added to the samples for 15 min at room temperature. The samples were centrifuged (13,500 rpm, 10 min, room temperature) to recover DNA. After that, 150 μL of TAE buffer (Tris-acetate-EDTA, pH 7.5) was added to each sample and incubated (50–60 °C, 10 min). Using primers synthesized by Integrated DNA Technologies Concentration, the total DNA was measured in a Nanodrop ND-1000 Spectrophotometer (Thermo Scientific, Waltham, MA, USA). Endpoint PCR was performed according to the manufacturer’s instructions (Taq Platinum, Invitrogen). Samples were submitted to electrophoresis in agarose gel for the bands to be visualized. PCR primers are listed in Table 1.

### 4.4. Vascular Reactivity

The mice were anesthetized by isoflurane inhalation (1–5% in 100% O_2_). After reducing the heartbeat and respiratory rate, the absence of painful stimulus was tested by pinching the animal’s foot. When the depth of anesthesia was confirmed, the mice were exsanguinated. The chest was exposed, and thoracic aortas were dissected and transferred to a Petri dish containing cold Krebs–Henseleit solution [mmol/L] (NaCl 130.00, NaHCO_3_ 14.9, C_6_H_12_O_6_ 5.5, KCl 4.7, KH_2_PO_4_ 1.18, MgSO_4_ 1.17, CaCl_2_ 1.6, HEPES 10.0; pH 7.4). Then, the fat and connective tissue surrounding the thoracic aorta was removed using a stereomicroscope, and then 2 mm long aortic rings were obtained. The aortic rings were positioned in a myograph chamber (Radnoti LLC, Covina, CA, USA) connected to a transducer system for recording isometric tension (PowerLab 8/35, ADInstruments, Colorado Springs, CO, USA). The aortic rings were stretched until they reached 0.75 g and maintained at this basal tension for 45 min in Krebs–Henseleit solution (pH 7.4, 37 °C). Then, we verified the presence of endothelium by the relaxation stimulated by ACh (10 µmol/L) in rings pre-contracted with phenylephrine (PE, 1 µmol/L). A 60% vasodilatory response to ACh was required to consider the presence of the endothelium in the preparations.

Cumulative concentration-response curves to NE (1 nmol/L to 10 µmol/L), ACh (0.1 µmol/L to 0.1 mmol/L), and KCl (0, 20, 40, 60, 80, and 100 mmol/L) were performed in aortic rings of *Gpc1^−/−^*, *Sdc1^−/−^*, C57BL/6 and CD1 in the absence or presence of heparinase III (15 mU/mL, 2 h, 37° C).

### 4.5. Western Blot

The thoracic aortas of all groups were cleaned, flash frozen in liquid nitrogen, and stored at −80 °C. The samples were macerated in RIPA buffer supplemented with protease cocktail inhibitor (Cat# P8340, Sigma Aldrich, St. Louis, MO, USA) and phosphatase inhibitors (NaF 1 mmol/L, Na3VO4 1 mmol/L, PMSF 10 mmol/L, Sigma Aldrich). The homogenates were centrifuged (4 °C, 13,000 rpm, 15 min), and the supernatants were used for protein quantification (Bradford assay). The samples (30 μg of protein) were loaded to a polyacrylamide gel (8 to 15%). Proteins were separated by electrophoresis and then transferred into a nitrocellulose membrane. The membranes were blocked with BSA 5% for one hour at room temperature, followed by the overnight incubation with primary antibodies (4 °C) against Sdc1, Sdc2, Sdc4, Gpc1, p-Akt Ser473, Akt total or β-actin. The membranes were then washed with TBS-T and incubated with a specific secondary antibody at room temperature for 60 min. β-actin was used as housekeeping control protein. The bands were detected by chemiluminescence using the LI-COR Odyssey Fc System and quantified using ImageJ Software, version 1.54 (NIH Image).

### 4.6. Statistical Analysis

The number of animals or the number of aortic rings that were used in this study is indicated by n.

In vascular reactivity studies, pD_2_ values were determined after negative logarithmic transformation of the normalized concentration-response curves, calculated as –log of the EC_50_. The maximum effect (Emax) values are considered as the maximum response achieved in the cumulative concentration curves for the relaxing or contracting agents. The area under the curve (AUC) was calculated for all concentration-response curves.

Results were expressed as the mean ± standard deviation (SD) of the obtained values (pD_2_, Emax, or AUC). Statistical analysis was performed using GraphPad Prism 8.0 software, using unpaired, two-tailed Student’s *t*-test, with Welch’s correction. Differences were considered statistically significant when *p* < 0.05.

## 5. Conclusions

Our studies indicate the physiological role of HS in the maintenance of the vascular tone in mice. This sheds light on the potential early mechanisms whereby the degradation of the glycocalyx and shedding of HS may contribute to the onset of endothelial dysfunction associated with vascular disease.

## Figures and Tables

**Table 1:** Primer sequences used for genotyping.

Primer Name	Forward	Reverse
*Syndecan 1 (Sdc1)*	CGC CGA AAC CTA CAG CCC TC	GCA TCG GCG AGT GGC GAG TC
*Neomycin*	CGAGACTAGTGAGACGTGCTACTTCC	
*Glypican 1 (Gpc1)* Mutant	AGCCGGCTTTTGTTGTCTC	CACGAGTGTGCTAGGATAGGG
*Glypican 1 (Gpc1)* Wild Type	CAGCGAAGTCCGCCAGAT	CAGACCTCCCGAGTGCTAGG

## Data Availability

The original contributions presented in this study are included in the article/Supplementary Material. Further inquiries can be directed to the corresponding author.

## Supplementary Materials

The following supporting information can be downloaded at: https://www.mdpi.com/article/10.3390/ijms27031386/s1.

## Abbreviations

The following abbreviations are used in this manuscript:

ACh	Acetylcholine
AUC	Area under the curve
BAECs	Bovine aortic endothelial cells
Emax	Maximum effect
eNOS	Endothelial nitric oxide synthase
GAGs	Glycosaminoglycans
Gpc1	Glypican 1
*Gpc1^−/−^*	Glypican 1 knockout mice
HS	Heparan sulfates
HSPGs	Heparan sulfate proteoglycans
KCl	Potassium chloride
NE	Norepinephrine
NO	Nitric oxide
pD_2_	Negative logarithm of the EC_50_ value
PE	Phenylephrine
SD	Standard deviation
Sdc1	Syndecan 1
*Sdc1^−/−^*	Syndecan 1 knockout mice
Sdc2	Syndecan 2
Sdc4	Syndecan 4
VSMC	Vascular smooth muscle cells
