# Supplementary material for: Heparan Sulfates Regulate Vascular Reactivity in Syndecan 1 Knockout Mice
Source: Int J Mol Sci. 2026 Jan 30;27(3):1386. doi: 10.3390/ijms27031386 (PMC12897610; doi:10.3390/ijms27031386)
Supplement: Supplementary file 1 [file ijms-27-01386-s001.zip › ijms-3669895-supplementary.pdf]

Minimal data set

- **Raw data from the concentration response curves performed on animal C57BL/6**

C57BL-6 Control - CURVE NE

Raw data

	7-19-18	7-20-18	7-21-18	7-26-18	7-30-18
**Log**	0,0011	0,0045	0,0016	0,0036	-0,0067
**-9,0000**	0,0012	0,0034	0,0018	0,0054	-0,0065
**-8,5000**	0,0217	0,0131	0,0188	0,0105	0,0102
**-8,0000**	0,0237	0,0140	0,0263	0,0204	0,0442
**-7,5000**	0,0589	0,0235	0,0678	0,0507	0,0797
**-7,0000**	0,1021	0,0637	0,0704	0,0639	0,1022
**-6,5000**	0,1139	0,0966	0,1526	0,0989	0,1324
**-6,0000**	0,1385	0,1092	0,1656	0,1196	0,1658
**-5,5000**	0,1646	0,1498	0,1783	0,1394	0,1731
**-5,0000**	0,1656	0,1512	0,1796	0,1398	0,1734

C57BL-6 Heparinase - CURVE NE

Raw data

	12-3-18	12-7-18 a	12-8-18	12-7-18 b	12-7-18
**Log**	-0,0006	0,0152	-0,0100	0,0013	-0,0090
**-9,0000**	0,0010	0,0019	0,0017	0,0045	0,0010
**-8,5000**	0,0070	0,0051	0,0063	0,0106	0,0124
**-8,0000**	0,0100	0,0102	0,0281	0,0108	0,0129
**-7,5000**	0,0216	0,0312	0,0415	0,0321	0,0331
**-7,0000**	0,0613	0,0792	0,0811	0,0716	0,0613
**-6,5000**	0,0813	0,1204	0,1293	0,1091	0,1031
**-6,0000**	0,0913	0,1229	0,1214	0,1209	0,1032
**-5,5000**	0,1030	0,1242	0,1217	0,1269	0,1029
**-5,0000**	0,1029	0,1264	0,1239	0,1272	0,1029

C57BL-6 Control - CURVE KCl

Raw data

[mol/L]	7-19-18	7-20-18	7-21-18	7-26-18	7-30-18
**0**	0,0071	0,0590	0,0031	0,0284	0,0177
**20**	0,1577	0,1548	0,1265	0,1981	0,2018
**40**	0,2267	0,2102	0,1706	0,2586	0,2481
**60**	0,2745	0,2561	0,1835	0,2791	0,2678
**80**	0,2778	0,2653	0,1935	0,2984	0,2955
**100**	0,2816	0,2679	0,2012	0,2991	0,2937

C57BL-6 Heparinase - CURVE KCl

Raw data

[mol/L]	12-3-18	12-7-18 a	12-7-18 b	12-7-18
**0,0000**	-0,0012	0,0017	0,0012	0,0011
**20,0000**	0,2353	0,2232	0,1824	0,1902
**40,0000**	0,2264	0,2374	0,2295	0,2194
**60,0000**	0,2463	0,2882	0,2373	0,2041
**80,0000**	0,2733	0,3001	0,2497	0,2170
**100,0000**	0,2632	0,3108	0,2471	0,1945

C57BL-6 Control - CURVE ACh

Raw data

	7-19-18	7-20-18	7-21-18	7-26-18	7-30-18
**Log**	0,1002	0,1354	0,1798	0,1002	0,1580
**-7,00000**	0,1001	0,1350	0,1767	0,0983	0,1556
**-6,50000**	0,0942	0,1260	0,1618	0,0959	0,1435
**-6,00000**	0,0902	0,1122	0,1592	0,0901	0,1402
**-5,50000**	0,0722	0,0845	0,1229	0,0709	0,1123
**-5,00000**	0,0502	0,0658	0,0987	0,0459	0,0841
**-4,50000**	0,0122	0,0341	0,0439	0,0218	0,0438
**-4,00000**	0,0022	0,0053	0,0073	0,0013	0,0147

C57BL-6 Heparinase - CURVE ACh

Raw data

	12-3-18	12-7-18 a	12-7-18 b	12-7-18 b	12-7-18
**Log**	0,0786	0,1780	0,2297	0,1124	0,1759
**-7,00000**	0,0783	0,1735	0,2234	0,1108	0,1752
**-6,50000**	0,0770	0,1680	0,2197	0,1043	0,1705
**-6,00000**	0,0690	0,1420	0,2086	0,0936	0,1615
**-5,50000**	0,0610	0,1097	0,1902	0,0744	0,1305
**-5,00000**	0,0582	0,0756	0,1701	0,0545	0,0817
**-4,50000**	0,0491	0,0492	0,1093	0,0335	0,0626
**-4,00000**	0,0430	0,0390	0,0824	0,0247	0,0411

- **Raw data from the concentration response curves performed on animal *Sdc1*$^{-/-}$**

Sdc-1 Control - CURVE NE

Raw data

	**11-09-18**	**11-16-18**	**11-26-18**	**11-29-18**
**Log**	0,0029	0,0070	0,0020	0,0011
**-9,0000**	0,0029	0,0092	0,0025	0,0014
**-8,5000**	0,0628	0,0170	0,0104	0,0026
**-8,0000**	0,0738	0,0395	0,0213	0,0479
**-7,5000**	0,1019	0,0695	0,0559	0,0639
**-7,0000**	0,1444	0,1085	0,1037	0,1085
**-6,5000**	0,2057	0,1491	0,1742	0,1677
**-6,0000**	0,2132	0,1890	0,2031	0,2113
**-5,5000**	0,2288	0,1888	0,2073	0,2175
**-5,0000**	0,2267	0,1884	0,2025	0,2212

Sdc-1 Heparinase - CURVE NE

Raw data

	**11-12-18**	**11-26-18**	**11-29-18**	**11-29-18b**
**Log**	-0,0022	0,0041	-0,0033	-0,0196
**-9,0000**	0,0010	0,0000	0,0028	-0,0119
**-8,5000**	0,0011	0,0047	0,0152	-0,0178
**-8,0000**	0,0135	0,0062	0,0165	-0,0101
**-7,5000**	0,0514	0,0402	0,0511	0,0801
**-7,0000**	0,0715	0,0662	0,0836	0,0919
**-6,5000**	0,1052	0,0715	0,0883	0,0902
**-6,0000**	0,1057	0,0755	0,0950	0,1050
**-5,5000**	0,1353	0,0744	0,0990	0,1068
**-5,0000**	0,1359	0,0738	0,1067	0,1097

Sdc-1 Control - CURVE KCl

Raw data

**[mol/L]**	**11-09-18a**	**11-5-18a**	**11-15-18b**	**11-30-18**
**0**	0,0035	0,0039	0,0091	0,0034
**20**	0,1352	0,1029	0,1759	0,2509
**40**	0,2035	0,1629	0,2188	0,2978
**60**	0,2348	0,2714	0,3022	0,3219
**80**	0,2547	0,2691	0,3285	0,3425
**100**	0,2535	0,2757	0,3217	0,3512

Sdc-1 Heparinase - CURVE KCl

Raw data

[mol/L]	11-2-18 b	11-2-18	11-5-18	11-12-18	11-15-18	11-16-18
**0,0000**	0,0375	0,0038	0,0164	0,0068	0,0152	-0,0065
**20,0000**	0,2499	0,1603	0,1982	0,2275	0,1957	0,1325
**40,0000**	0,2722	0,1603	0,2320	0,2314	0,1895	0,1693
**60,0000**	0,3409	0,1951	0,2591	0,2727	0,2408	0,2162
**80,0000**	0,3555	0,2362	0,2885	0,2597	0,2826	0,2447
**100,0000**	0,3819	0,2309	0,2795	0,2574	0,2869	0,2334

Sdc-1 Control - CURVE ACh

Raw data

	11-5-18	11-9-18	11-12-18	11-15-18	11-16-18
**Log**	0,1355	0,1702	0,1700	0,1012	0,1270
**-7,00000**	0,1350	0,1701	0,1673	0,1011	0,1246
**-6,50000**	0,1332	0,1698	0,1628	0,0988	0,1194
**-6,00000**	0,1255	0,1571	0,1506	0,0878	0,1095
**-5,50000**	0,1044	0,1390	0,1312	0,0749	0,0934
**-5,00000**	0,0835	0,1019	0,0921	0,0483	0,0744
**-4,50000**	0,0672	0,0789	0,0712	0,0323	0,0543
**-4,00000**	0,0465	0,0596	0,0684	0,0240	0,0333

Sdc-1 Heparinase - CURVE ACh

Raw data

	11-2-18 b	11-5-18	11-12-18	11-16-18	11-26-18
**Log**	0,2664	0,1767	0,0909	0,0863	0,0987
**-7,00000**	0,2643	0,1762	0,0899	0,0861	0,0980
**-6,50000**	0,2552	0,1608	0,0882	0,0816	0,0944
**-6,00000**	0,2451	0,1504	0,0824	0,0765	0,0844
**-5,50000**	0,2325	0,1401	0,0777	0,0654	0,0763
**-5,00000**	0,1810	0,1200	0,0615	0,0598	0,0653
**-4,50000**	0,1517	0,0919	0,0536	0,0558	0,0541
**-4,00000**	0,1321	0,0810	0,0504	0,0508	0,0461

- **Raw data from the concentration response curves performed on animal CD1**

CD-1 Control - CURVE NE
Raw data

	7-16-18	7-17-18	7-18-18	7-24-18
**Log**	0,0048	0,0106	0,0024	-0,0006
**-9,0000**	0,0035	0,0179	0,0166	0,0084
**-8,5000**	0,0252	0,0228	0,0251	0,0544
**-8,0000**	0,0447	0,0822	0,1428	0,1065
**-7,5000**	0,0905	0,1501	0,1805	0,1747
**-7,0000**	0,1545	0,1805	0,2255	0,2441
**-6,5000**	0,2144	0,2576	0,2742	0,2856
**-6,0000**	0,2645	0,3037	0,3005	0,3348
**-5,5000**	0,2848	0,3264	0,3392	0,3458
**-5,0000**	0,2847	0,3318	0,3458	0,3542

CD-1 Heparinase - CURVE NE
Raw data

	10-12-18	10-11-18	10-5-18	9-24-18
**Log**	0,0006	0,0035	0,0029	0,0171
**-9,0000**	0,0091	0,0051	0,0041	0,0038
**-8,5000**	0,0091	0,0398	0,0027	0,0285
**-8,0000**	0,0079	0,0337	0,1485	0,0717
**-7,5000**	0,0476	0,1526	0,2148	0,1036
**-7,0000**	0,2276	0,1829	0,2148	0,1478
**-6,5000**	0,2797	0,2227	0,2348	0,1639
**-6,0000**	0,2831	0,2132	0,2242	0,1872
**-5,5000**	0,2593	0,2177	0,2417	0,1965
**-5,0000**	0,2604	0,2197	0,2567	0,1943

CD-1 Control - CURVE KCl
Raw data

[mol/L]	7-17-18	7-18-18	7-24-18	7-25-18	10-03-19d	10-03-19a	12-03-19a
**0**	0,0067	0,0507	0,0291	0,0121	0,023	0,0164	0,0114
**20**	0,2217	0,2334	0,2594	0,1964	0,2522	0,2163	0,2655
**40**	0,2934	0,2613	0,3291	0,2563	0,31882	0,2815	0,3141
**60**	0,3482	0,3086	0,3694	0,3277	0,3636	0,3657	0,3785
**80**	0,3553	0,3475	0,3703	0,3611	0,4012	0,4017	0,4222
**100**	0,3639	0,3526	0,3702	0,3678	0,4101	0,4042	0,4256

CD-1 Heparinase - CURVE KCl

Raw data

[mol/L]	10-18-18	10-12-18	10-11-18	10-8-18	10-5-18
**0,0000**	-0,0100	0,0537	-0,0237	-0,0129	0,0181
**20,0000**	0,2763	0,2159	0,2059	0,2169	0,2308
**40,0000**	0,2825	0,2814	0,2505	0,2145	0,3339
**60,0000**	0,3657	0,3476	0,3088	0,2659	0,3462
**80,0000**	0,3829	0,3704	0,3136	0,3434	0,3809
**100,0000**	0,3815	0,3600	0,3119	0,3293	0,3807

CD-1 Control - CURVE ACh

Raw data

	8-1-18	9-24-18	10-4-18	10-11-18	10-18-18
**Log**	0,3202	0,2894	0,2887	0,3484	0,2122
**-7,00000**	0,3198	0,2890	0,2878	0,3434	0,2120
**-6,50000**	0,3068	0,2774	0,2716	0,3356	0,2027
**-6,00000**	0,2899	0,2376	0,2273	0,2766	0,1581
**-5,50000**	0,2302	0,1756	0,1652	0,2136	0,1123
**-5,00000**	0,1502	0,0823	0,0807	0,1002	0,0841
**-4,50000**	0,0919	0,0395	0,0589	0,0604	0,0232
**-4,00000**	0,0024	0,0116	0,0191	0,0102	0,0048

CD-1 Heparinase - CURVE ACh

Raw data

	10-18-18	10-12-18	10-11-18	10-8-18	10-5-18
**Log**	0,1556	0,2614	0,1547	0,2600	0,3322
**-7,00000**	0,1514	0,2562	0,1539	0,2578	0,3318
**-6,50000**	0,1408	0,2467	0,1530	0,2567	0,3314
**-6,00000**	0,1404	0,2063	0,1413	0,2471	0,3022
**-5,50000**	0,1042	0,1607	0,1089	0,2148	0,2723
**-5,00000**	0,0835	0,1241	0,0952	0,1219	0,2411
**-4,50000**	0,0644	0,0927	0,0820	0,1224	0,2116
**-4,00000**	0,0423	0,0856	0,0714	0,0956	0,1725

- **Raw data from the concentration response curves performed on animal *Gpc1*$^{-/-}$**

Gpc-1 Control - CURVE NE

Raw data

	9-27-18	9-07-18	10-25-18	10-29-18
**Log**	0,0147	0,0126	0,0110	0,0051
**-9,0000**	0,0148	0,0108	0,0110	0,0140
**-8,5000**	0,0208	0,0125	0,0232	0,0208
**-8,0000**	0,0289	0,0183	0,0297	0,0246
**-7,5000**	0,0933	0,0816	0,0775	0,0557
**-7,0000**	0,1249	0,1311	0,1053	0,1129
**-6,5000**	0,1533	0,1705	0,1961	0,1708
**-6,0000**	0,1885	0,1984	0,2058	0,1978
**-5,5000**	0,1986	0,2159	0,2125	0,2056
**-5,0000**	0,2024	0,2245	0,2117	0,2072

Gpc-1 Heparinase - CURVE NE

Raw data

	8-10-18	9-07-18	9-27-18	10-19-18	10-29-18
**Log**	0,013	0,0083	0,0024	0,0085	0
**-9,0000**	0,0071	0,0112	0,015	0,0045	0
**-8,5000**	0,013	0,0131	0,0168	0,0181	0,0038
**-8,0000**	0,0249	0,0368	0,0269	0,0209	0,0166
**-7,5000**	0,0728	0,0693	0,0683	0,0525	0,0712
**-7,0000**	0,1089	0,114	0,1372	0,1085	0,1522
**-6,5000**	0,1536	0,1693	0,19625	0,13013	0,2211
**-6,0000**	0,1978	0,1545	0,2369	0,160051	0,2434
**-5,5000**	0,2124	0,1687	0,24726	0,18043	0,2515
**-5,0000**	0,2196	0,1665	0,245	0,18074	0,2563

Gpc-1 Control - CURVE KCl

Raw data

[mol/L]	10-19-18	8-10-18	9-7-18	08-25-18
**0**	0,0176	0,0230	0,0069	0,0202
**20**	0,1405	0,1981	0,1567	0,1683
**40**	0,1942	0,2706	0,2433	0,2342
**60**	0,2689	0,2979	0,3553	0,3212
**80**	0,2879	0,3061	0,3681	0,3355
**100**	0,2717	0,3120	0,3597	0,3421

Gpc-1 Heparinase - CURVE KCl

Raw data

[mol/L]	8-10-18	9-27-18	10-19-18	10-25-18
0,0000	-0,0080	0,0344	0,0096	0,0180
20,0000	0,2002	0,1993	0,1580	0,1685
40,0000	0,2396	0,2806	0,1722	0,1792
60,0000	0,2577	0,3056	0,2373	0,2169
80,0000	0,2694	0,3249	0,2577	0,2354
100,0000	0,2774	0,3715	0,2507	0,2343

Gpc-1 Control - CURVE ACh

Raw data

	8-10-18	10-19-18	10-29-18	10-25-18	9-7-18
Log	0,1102	0,0904	0,0791	0,0854	0,0582
-7,00000	0,1100	0,0902	0,0789	0,0850	0,0579
-6,50000	0,1006	0,0894	0,0771	0,0844	0,0566
-6,00000	0,0951	0,0795	0,0642	0,0756	0,0550
-5,50000	0,0840	0,0625	0,0519	0,0584	0,0404
-5,00000	0,0656	0,0484	0,0326	0,0415	0,0296
-4,50000	0,0463	0,0309	0,0232	0,0342	0,0258
-4,00000	0,0311	0,0232	0,0211	0,0250	0,0224

Gpc-1 Heparinase - CURVE ACh

Raw data

	9-27-18	10-19-18	10-25-18	10-25-18	10-29-18
Log	0,1133	0,0832	0,4095	0,1841	0,2310
-7,00000	0,1155	0,0817	0,4018	0,1888	0,2389
-6,50000	0,1093	0,0716	0,4028	0,1804	0,2219
-6,00000	0,1061	0,0602	0,3915	0,1502	0,2172
-5,50000	0,0828	0,0401	0,3307	0,1207	0,1608
-5,00000	0,0560	0,0487	0,2805	0,0840	0,1228
-4,50000	0,0407	0,0257	0,2102	0,0409	0,0727
-4,00000	0,0302	0,0159	0,1905	0,0307	0,0524

- **Raw data obtained from western blot bands through pixel measurements using scion image software**

	Syndecan-1	Actin
1 C57	9.143,962	15924,134
2 C57	12.537,962	16136,426
3 C57	12.693,255	17356,305
4 C57	15.051,497	16544,305
5 C57	12.037,790	17578,719
1 Sdc-1	142,113	17240,669
2 Sdc-1	110,820	17154,012
3 Sdc-1	153,920	18546,255
4 Sdc-1	195,385	16086,962
5 Sdc-1	184,113	14634,841

	Syndecan-1	Actin
1 CD-1	4879,355	17.201,184
2 CD-1	4315,305	17.850,598
3 CD-1	5972,841	19.391,477
4 CD-1	5292,184	17.130,841
5 CD-1	4893,305	15.798,891
1 Gpc-1	15853,740	17.271,719
2 Gpc-1	16669,326	16.763,426
3 Gpc-1	19421,719	17.369,426
4 Gpc-1	15583,841	15.145,305
5 Gpc-1	14325,740	12.765,891

	Syndecan-2	Actin
1 C57	1999,770	17813,790
2 C57	1892,234	17496,962
3 C57	2664,770	17222,376
4 C57	2700,355	18358,548
5 C57	3844,891	17399,548
1 Sdc-1	14955,548	14795,719
2 Sdc-1	17840,376	14722,305
3 Sdc-1	18124,790	14766,184
4 Sdc-1	17232,841	15336,426
5 Sdc-1	17600,497	14014,790

	Syndecan-2	Actin
1 CD-1	6.005,962	13.271,589
2 CD-1	6.158,841	16.714,447
3 CD-1	6.330,255	13.579,962
4 CD-1	6.484,083	14.294,962
5 CD-1	7.716,841	12.105,255
1 Gpc-1	15.071,255	10.207,184
2 Gpc-1	15.259,669	11.703,598
3 Gpc-1	15.390,669	11.257,184
4 Gpc-1	14.470,962	12.514,062
5 Gpc-1	13.543,962	11.571,598

	Syndecan-4	Actin
1 C57	1802,527	18131,962
2 C57	1953,770	17737,548
3 C57	2025,355	19040,962
4 C57	2367,648	16954,841
5 C57	2117,234	18416,548
1 Sdc-1	15585,962	18373,719
2 Sdc-1	15358,962	17223,962
3 Sdc-1	15410,083	18554,548
4 Sdc-1	11302,184	14798,598
5 Sdc-1	15417,548	17995,669

	Syndecan-4	Actin
1 CD-1	3.499,891	17.477,962
2 CD-1	5.221,548	18.026,255
3 CD-1	2.852,669	18.312,841
4 CD-1	3.495,891	17.762,548
5 CD-1	3.702,305	17.839,669
1 Gpc-1	13.523,376	15.926,548
2 Gpc-1	14.816,376	15.088,548
3 Gpc-1	14.613,962	15.342,548
4 Gpc-1	14.235,548	15.377,790
5 Gpc-1	12.409,790	14.605,719

	Glypican-1	Actin
1 - C57	14311,083	11970,719
2 - C57	14266,134	13688,740
3 - C57	13982,719	12761,134
4 - C57	16015,426	15205,548
5 - C57	15030,841	11118,719
1 - Syn-1	2365,648	10933,134
2 - Syn-1	1763,355	13607,376
3 - Syn-1	4118,770	11653,719
4 - Syn-1	6850,083	13086,305
5 - Syn-1	7354,083	11279,426

	Glypican-1	Actin
1 - CD-1	7.707,134	18.537,376
2 - CD-1	12.017,255	18.474,012
3 - CD-1	13.387,790	16.386,548
4 - CD-1	14.737,669	17.394,841
5 - CD-1	13.329,669	16.341,184
1 - Gpc-1	163,950	16.617,719
2 - Gpc-1	159,243	18.849,841
3 - Gpc-1	261,536	17.969,841
4 - Gpc-1	159,013	18.288,134
5 - Gpc-1	103,456	19.055,790

C57BL/6

	p-Akt		Akt total		Actin
1	14.372,548	1	14.314,548	1	9.275,062
2	15.293,083	2	16.774,376	2	9.087,355
3	16.432,962	3	18.145,841	3	9.151,941
4	16.097,426	4	16.219,255	4	10.343,355
5	14.914,912	5	16.136,790	5	8.786,012

Sdc1$^{-/-}$

	p-Akt		Akt total		Actin
6	4.885,841	6	6.496,841	6	6.417,669
7	5.264,719	7	6.240,305	7	5.812,477
8	5.980,255	8	7.357,841	8	6.373,284
9	6.494,719	9	7.691,134	9	8.770,719
10	5.046,719	10	6.551,477	10	6.723,941

CD-1

	p-Akt		Akt total	Actin
1	10.964,719	1	26.576,075	17.252,477
2	11.164,962	2	26.759,681	17.635,477
3	10.507,548	3	20.876,388	17.427,012
4	11.859,305	4	22.620,782	17.395,305
5	10.384,083	5	22.289,560	17.458,376

Gpc1 $^{-/-}$

	p-Akt		Akt total	Actin
6	6.254,548	6	20.353,146	15.149,426
7	6.619,548	7	21.177,267	15.448,376
8	6.233,962	8	19.249,903	17.121,719
9	5.440,477	9	17.092,539	17.653,719
10	6.728,962	10	16.554,317	17.769,548
